# Does 5-ALA Fluorescence Microscopy Improve Complete Resectability in Cerebral/Cerebellar Metastatic Surgery? A Retrospective Data Analysis from a Cranial Center

**DOI:** 10.3390/cancers16122242

**Published:** 2024-06-17

**Authors:** Hraq Mourad Sarkis, Samer Zawy Alsofy, Ralf Stroop, Marc Lewitz, Stephanie Schipmann, Markus Unnewehr, Werner Paulus, Makoto Nakamura, Christian Ewelt

**Affiliations:** 1 Department of Medicine, Faculty of Health, Witten/Herdecke University, 58448 Witten, Germany; hraq@hotmail.com (H.M.S.); ralf@stroop.de (R.S.); mlewitz@barbaraklinik.de (M.L.); munnewehr@barbaraklinik.de (M.U.); 2 Department of Neurosurgery, St. Barbara-Hospital, Academic Hospital of Westfaelische Wilhelms-University Muenster, 59073 Hamm, Germany; cewelt@barbaraklinik.de; 3 Department of Neurosurgery, St. Marien-Hospital, Academic Hospital of the Hannover Medical School, 49076 Osnabrueck, Germany; 4 Department of Neurosurgery, University Hospital Muenster, 48149 Muenster, Germany; stephanie.schipmann@googlemail.com; 5 Institute of Neuropathology, University Hospital Muenster, 48149 Muenster, Germany; werner.paulus@uni-muenster.de; 6 Department of Neurosurgery, Academic Hospital Koeln-Merheim, Witten/Herdecke University, 51109 Koeln, Germany; nakamuram@kliniken-koeln.de

**Keywords:** 5-aminolevulinic acid (5-ALA), fluorescence, metastatic brain tumors, malignant gliomas, protoporphyrin IX

## Abstract

**Simple Summary:**

In the present study, the intraoperative fluorescence of brain metastases after the administration of 5-aminolevulinic acid (5-ALA) is investigated in 80 cases. Brain metastases fluoresced in 57.5% of cases, with no significant correlation between fluorescence and primary tumor or histological subtype. Complete resection of brain metastases was detected in 82.5%, of which 56.1% were fluorescence positive, compared to 43.9% which were non-fluorescent. Thus, prior administration of 5-ALA tended to improve the resectability rate by 12.1%. Fluorescence-positive and -negative metastases showed significantly different overall survival in this study. Therefore, administration of 5-ALA as a surgical adjuvant may be beneficial in resecting brain metastases and may potentially optimize the surgical procedure.

**Abstract:**

(1) Background: In this study, the intraoperative fluorescence behavior of brain metastases after the administration of 5-aminolevulinic acid (5-ALA) was analyzed. The aim was to investigate whether the resection of brain metastases using 5-ALA fluorescence also leads to a more complete resections and thus to a prolongation of survival; (2) Methods: The following variables have been considered: age, sex, number of metastases, localization, involvement of eloquent area, correlation between fluorescence and primary tumor/subtype, resection, and survival time. The influence on the degree of resection was determined with a control MRI within the first three postoperative days; (3) Results: Brain metastases fluoresced in 57.5% of cases. The highest fluorescence rates of 73.3% were found in breast carcinoma metastases and the histologic subtype adenocarcinoma (68.1%). No correlation between fluorescence behavior and localization, primary tumor, or histological subtype was found. Complete resection was detected in 82.5%, of which 56.1% were fluorescence positive. There was a trend towards improved resectability (increase of 12.1%) and a significantly longer survival time (*p* = 0.009) in the fluorescence-positive group; (4) Conclusions: 5-ALA-assisted extirpation leads to a more complete resection and longer survival and can therefore represent a low-risk addition to modern surgery for brain metastases.

## 1. Introduction

Secondary or metastatic brain tumors are the most common malignancies of the central nervous system (CNS). According to population studies, the estimated prevalence of brain metastases in the US is 7–14 cases per 100,000 people [1]. In patients with previously diagnosed cancer, 10–30% will also develop brain metastasis [2]. It is important to note that the spectrum of metastatic primary cancers and the risk of CNS involvement vary according to patient age [3,4]. For example, CNS metastases occur more frequently in adults, with the highest incidence in the fifth to seventh decades of life [3,5]. In children, leukemia is the most common cause of brain metastasis, followed by lymphoma and cancer of the bone/soft tissue [3]. Cancers of the lung, breast, kidney, gastrointestinal tract, and skin are the most common primary causes of brain metastases in adults. However, they can also result from another part of the body [6,7].

Brain metastases are manifested by headache (50%), neurological failure symptoms (50%), organic brain psychosyndrome (30%), epileptic seizures (15–20%) and other signs of intracranial pressure (nausea, vomiting). Depending on the location, impaired vision or consciousness can also occur [8]. Magnetic resonance imaging (MRI), with and without gadolinium, in different sequences (MR perfusion imaging (MRP), diffusion-weighted imaging (DWI), cerebral blood volume (rCBV)) are used as the gold standard for the initial assessment and are more advantageous than the other available diagnostic imaging [9,10].

Current treatment of cerebral/cerebellar brain metastases is based on many prognostic factors, such as Karnofsky Performance Status (KPS), tumor histology, number of metastases (singular, solitary or multiple), age of the patient, status of the systemic disease (extracranial metastasis), time of occurrence of the brain metastasis in relation to the initial diagnosis of the primary tumor (synchronous, metachronous), previous pretreatment and localization/resectability of the tumor in the brain [11,12]. The therapeutic approaches include surgical resection with/without subsequent whole-brain radiation (WBRT), stereotactic radiosurgery (SRS) and chemotherapy. The standard for treatment of these lesions is usually surgery and/or radiosurgery.

The controlled systemic disease is of great importance for the prognosis of brain metastasis. Because of this, Sperduto et al. was proposed a new score, Graded Prognostic Assessment (GPA), in which the extracranial metastases were taken into account compared to the Recursive Partitioning Analysis (RPA). This score was updated in 2012 (Table 1) [13,14].

Furthermore, the response assessment in neuro-oncology criteria for brain metastases (RANO-BM), established in 2015, categorizes tumor response into four distinct types based on imaging techniques such as MRI or CT, along with clinical features. These response types are as follows: complete response, partial response, stable disease, and progression. This classification helps in precisely assessing the effectiveness of treatment and guiding clinical decisions based on observable changes in tumor status and patient condition [15].

5-Aminolevulinic Acid (5-ALA)

The amino acid belongs to the γ-ketocarboxylic acids and is an intermediate product of the porphyrin synthesis and thus an anabolite of the heme biosynthesis [16]. These syntheses produce the intermediate protoporphyrin IX (PpIX), which has fluorescent properties. The fluorescence can be seen under ultraviolet light (UV) with a wavelength of 375–440 nm [17]. PpIX accumulates more in tumor cells compared to normal tissue. This could be due, among other things, to a change in the permeability of the blood–brain barrier in the area of the tumor to 5-ALA and the reason why normal brain tissue is largely protected from 5-ALA resorption [18]. In addition, increased activity of 5-ALA dehydratase could be causative for accumulation of PpIX, as it produces more porphobilinogen (PBG), the precursor of PpIX [19]. Furthermore, decreased activity of iron chelatase or an overall lower available amount of iron in malignant cells could be for accumulation of the fluorescent metabolite, as the final steps of heme synthesis stagnate or only occur under more difficult conditions when PpIX accumulates [20]. Stepp et al. have summarized the possible reasons for tumor-selective PpIX accumulation in a table (Table 2) [21].

Stummer et al. found in 1998 that after exogenous administration of 5-ALA and due to the defective blood–brain barrier in human malignant gliomas, fluorescent PpIX accumulated and was checked intraoperatively in an ultraviolet light with a wavelength of 375–440 nm with a neuropathological–histological examination Salmon red specificity of 100% and a sensitivity of 85% (n = 89) [17,18]. It is not only gliomas, but also benign tumors of the dura mater (meningiomas) that can show an increased accumulation and thus also intraoperative fluorescence of PpIX [22]. Marbacher et al. reported in a study from 2014 that among the metastases the highest percentage (48%) of 5-ALA-positive fluorescence was found in adenocarcinomas. Overall, positive intraoperative 5-ALA fluorescence could be documented in 52% (n = 34/65) of the patients with brain metastases [23]. A higher 5-ALA fluorescence rate was demonstrated in the following studies: in a paper from 2012, Kamp et al. described a positive fluorescence in 62% of the metastases; 23 of 33 (70%) adenocarcinomas were positive [24]. In a series of 11 brain metastases, Utsuki et al. reported a 5-ALA positivity of 82% [25].

The purpose of the work

The aim of this work is to verify whether positive 5-ALA fluorescence-guided metastasectomy has an impact on the extent of resection, and thus, whether it may lead to a prolongation of median survival. Based on the available data regarding the use of 5-ALA in glioblastoma surgery, the question was whether 5-ALA-guided metastatic surgery can also provide better tumor delineation and thus better quality of life, as tumor margins can be better visualized using 5-ALA in glioblastoma surgery, thus avoiding postoperative neurological deficits in addition to the use of intraoperative electrophysiological monitoring and intraoperative imaging techniques.

## 2. Materials and Methods

### 2.1. Ethics Vote

For our scientific work, an ethics vote according to the Ethics Committee of the Medical Association of Westphalia-Lippe and the Westphalian Wilhelms University of Münster was approved (approval code: 2019-226-f-S).

### 2.2. Inclusion of Patients

This work is a retrospective study. This study included 73 patients who were treated between 1 January 2017 and 1 January 2020 with a diagnosis or suspected diagnosis of a brain metastasis in our institute. The data collection lasted until April 2022. The following exclusion criteria applied: patients < 18 years of age, patients with suspected glial brain tumor, meningioma, cerebral lymphoma, and brain abscesses as well as other inflammatory intracerebral lesions. The clinical exclusion criteria for the administration of 5-ALA were the following: patients with a metabolic disease of heme biosynthesis (porphyria), patients with a history of photosensitivity and patients during pregnancy. A preoperative detailed explanation of treatment options, risks and complications, benefits and side effects of the administration of 5-ALA for fluorescence-guided resection was carried out.

### 2.3. Data Acquisition

The following data were determined: age, gender, final diagnosis, tumor location, type of primary tumor, histological subtype, number of metastases, 5 ALA fluorescence (yes, no), extent of postoperative resection, and laboratory parameters such as liver function tests, renal function tests, and complete blood count.

### 2.4. Preoperative Preparation

On the morning of the tumor resection, the patients received an oral dose of 5-ALA at a dose of 20 mg/kg body weight between 3 and 4 h before induction of anesthesia [26]. To produce the 5-ALA solution with a concentration of 30 mg/mL, 1.5 g of 5-ALA HCl powder are dissolved in 50 mL of drinking water solution and administered orally in an application quantity of 20 mg/kg of body weight (Gliolan^®^, Medac GmbH, Wedel/Tornesch, Germany [26], or 5-ALA HCl MedicalOrder^®^ Center, Ahlen, Germany). Antiedematous therapy was administered preoperatively using dexamethasone 4 mg three times a day.

### 2.5. Intraoperative Procedure

**Figure 1:**
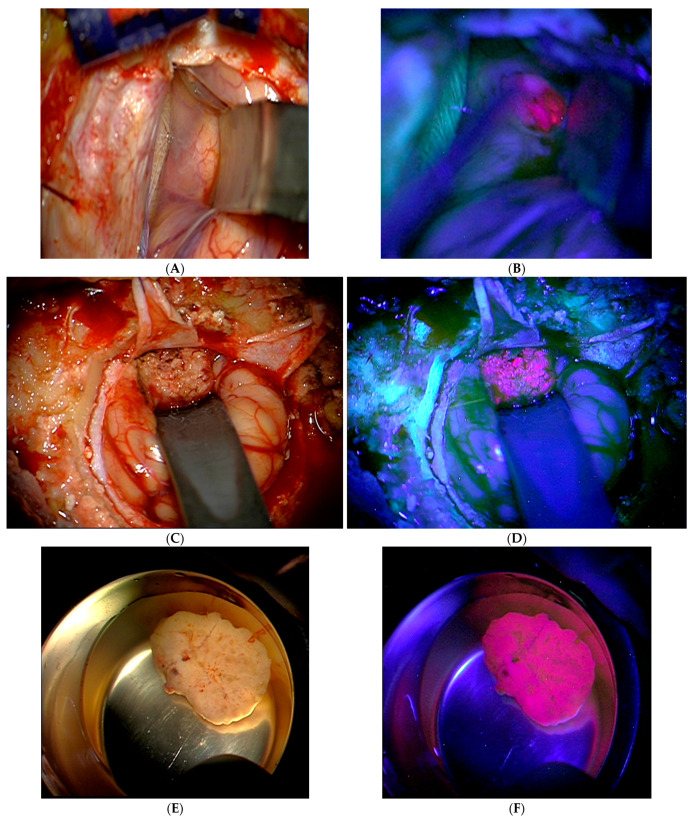
Intraoperative OP microscopic tumor visualization using 5-ALA. These images, which were taken after 5-ALA administration, show the sites under normal light conditions (**A**,**C**,**E**) and illuminated with UV light (**B**,**D**,**F**),which provided a strong, lava-like 5-ALA fluorescence.

The metastases were resected according to the usual microneurosurgical procedure. The resections were divided into partial resection and total resection of the metastases. The supramarginal resection, to a depth of about 5 mm, was carried out in cases with total resection of the metastasis, in which the metastasis was not in eloquent areas and as long as intraoperative neuromonitoring did not reveal any pathological changes during the resection. In the case of fluorescent metastases, the metastases were resected in repeated alternation between white light and UV light imaging. The undetectable, or attenuated fluorescence was defined as complete resection. The 5-ALA fluorescence of a metastasis was assessed intraoperatively by the neurosurgeon and documented in the surgical report as absent, weak or strong fluorescence (Figure 1).

### 2.6. Processing of the Material

Macroscopic, histopathological and immunohistochemical examinations were performed at the Neuropathological Institute of the University Hospital Münster to confirm the diagnosis of the tumor tissue removed intraoperatively.

### 2.7. Postoperative Evaluation and Follow-Up

After the surgery, patients were closely monitored for at least one night in the surgical intensive care unit, and exposure to strong light or sunlight was avoided for 24 h due to phototoxicity. Sun exposure was also avoided by the nursing staff because of possible skin sensitization. Both preoperatively and 24 h postoperatively, liver and kidney function values were measured and the complete blood count was determined. To evaluate the extent of the resection, an MRI of the skull with and without contrast was acquired within the first three days postoperatively. During the stay, the preoperative anti-edematous therapy was tapered off within 5 days postoperatively. After receiving the histopathological findings, the patients were presented in an interdisciplinary tumor board and classified for an appropriate therapy, which was initiated after patient consensus. The first follow-up by magnetic resonance imaging of the skull, with and without contrast, was recommended 3 months postoperatively.

### 2.8. Substance and Devices

- Gliolan 30 mg/mL powder for the preparation of a solution with a dosage of 20 mg/kg.
- Microsurgical tumor resection was performed using a surgical microscope with support for intraoperative visualization of fluorescent tumor tissue (OPMI^®^ Pentero^®^ 800 with BLUE^®^ 400 function, Zeiss, Oberkochen, Germany).
- Magnetic resonance imaging was performed with a 1.5 Tesla MRI scanner (MAGNE-TOM^®^, Siemens, Munich, Germany) to assess the extent of the resection and as a postoperative follow-up.

### 2.9. Software and Program

The data were taken from the patient files, general practitioner reports, OP reports, explanations and histopathological findings sent by the Institute for Neuropathology, the electronic patient files (Orbis^®^, Dedalus Healthcare GmbH, Bonn, Germany), the intensive care chart (MetaVision Suite^®^, iMDsoft, Düsseldorf, Germany), the radiological diagnostics of the company’s Medavis program (JiveX DICOM Viewer^®^, VISUS Health IT GmbH, Bochum, Germany), as well as the transfer letters from earlier hospitals and summarized pseudonymously in tables and diagrams, which were created with the help of the spreadsheet program (Excel^®^, Microsoft, Irvine, CA, USA).

### 2.10. Statistical Analysis

The statistical analysis was carried out using a two-sample t-test (Excel^®^, Microsoft, CA, USA) with dependent samples (pair comparison test) in order to determine the statistical probability of a correlation between pre- and postoperative laboratory chemical changes after administration of 5-ALA. The chi-square test from SPSS (Statistical Package for the Social Sciences, SPSS^®^ 27.0, IBM, Ehningen, Germany) was used to check various possible influencing factors on the 5-ALA fluorescence. The Kaplan–Meier survival curve was used to compare a group that received 5-ALA-guided metastatic resection and a group that received conventional resection. The *p*-value < 0.05 was considered statistically significant.

## 3. Results

A total of 80 5-ALA-assisted operations were performed on 73 patients with brain metastases; 7 patients underwent a second operation to resect a brain metastasis in another localization. 5-ALA fluorescence was observed in 57.5% of brain metastases, while 42.5% showed no 5-ALA fluorescence. The mean age was 63 years (age range: 33–83 years). In total, 38 (52.1%) were male and 35 (47.9%) were female. The male to female ratio was (1.1:1). Of the whole patient collective, 53 (72.6%) patients had solitary or singular brain metastases and 20 (27.4%) patients had multiple brain metastases. The metastases were located in different locations in the following percentages: 75% supratentorial, 21% infratentorial, and 4% supra/infratentorial. In 49% of the cases (n = 39/80), the brain metastases were located in an eloquent area and 51% (n = 41/80) were in non-eloquent areas. Statistically, no correlation was observed between the location of supra/infratentorial brain metastasis and 5-ALA fluorescence (*p* = 0.391; Chi-square test, SPSS), nor between the localization of brain metastasis in eloquent/non-eloquent areas and the extent of 5-ALA fluorescence (*p* = 0.279; Chi-square test, SPSS). The overall disease- and patient-related characteristics are summarized in Table 3.

### 3.1. Laboratory Values

The creatinine values, hemoglobin level in the serum, and the platelets were significantly lower on the first postoperative day compared to the preoperative values (*p* = 0.0001, *p* = 4.35 × 10^−9^, *p* = 3.19 × 10^−17^). Leukocytes as well as GPT values were significantly higher on the first postoperative day than preoperative (*p* = 0.0005, *p* = 1.96 × 10^−6^). The G-GT values showed no significant difference between the pre- and post-operative measurements (*p* = 0.2).

### 3.2. The Incidence of the Primary Tumor and Subtypes

Regarding the primary tumor, 46 (57.5%) patients were diagnosed with a bronchial carcinoma (A non-small cell lung carcinoma (NSCLC) in 38 patients and small cell lung cancer (SCLC) in 8 patients), 15 (18.8%) patients with breast cancer, 9 (11.3%) with gastrointestinal carcinoma, 5 (6.3%) with renal cell carcinoma, 4 (5.0%) with malignant melanoma, and 1 patient (1.3%) with a gynecological tumor.

Regarding the histological subtypes, 47 patients (58.7%) suffered from adenocarcinoma, 10 (12.5%) from squamous cell carcinoma, 8 (10%) from small cell carcinoma, 4 (5.0%) from clear cell carcinoma, 4 (5.0%) from malignant melanoma, and 2 (2.5%) from giant cell carcinoma. Furthermore, following histopathological examination of the tissue, five (6.3%) patients exhibited poorly differentiated brain metastases from carcinoma.

### 3.3. Correlation between the 5-ALA Fluorescence and the Primary Tumor

**Figure 2:**
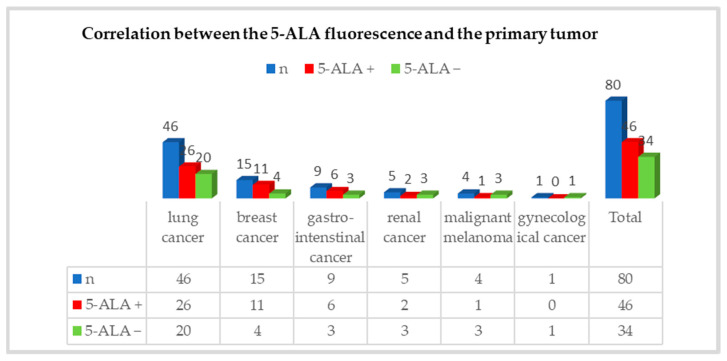
Correlation between 5-ALA fluorescence and primary tumor. Blue bars show the total number of cases; red bars = 5-ALA positive cases; green bars = 5-ALA negative cases.

In this study, 46 (57.5%) brain metastases showed either strong or weak 5-ALA fluorescence intraoperatively and 34 brain metastases (42.5%) showed no 5-ALA fluorescence. Bronchial carcinomas were causative of cerebral/cerebellar metastasis in 57.5% (n = 46/80). The fluorescence rate from bronchial carcinoma was 56.5% (n = 26/46). Furthermore, the fluorescence rate was 73.3% (n = 11/15) in brain metastases from breast carcinoma and 66.6% (n = 6/9) in brain metastases from tumors originating in the gastrointestinal tract. Further information about the correlation between 5-ALA fluorescence and primary tumor is presented in Figure 2. However, this study shows no statistical correlation between 5 ALA fluorescence and the primary tumor (*p* = 0.50; Chi-square test, SPSS).

### 3.4. Correlation between the 5-ALA Fluorescence and the Histological Subtype

**Figure 3:**
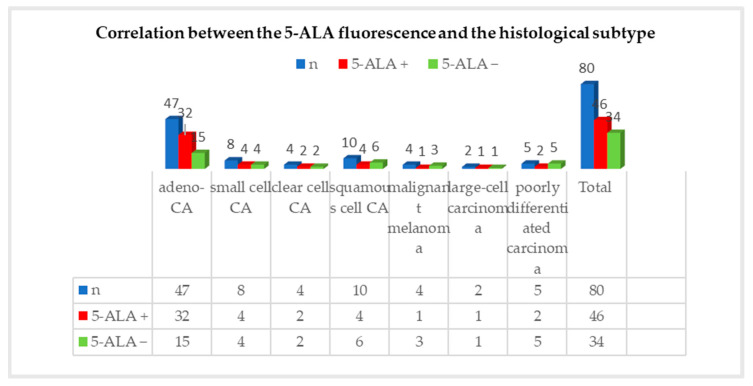
Correlation between 5-ALA fluorescence and histological subtype. Blue bars show the total number of cases; red bars = 5-ALA positive cases; green bars = 5-ALA negative cases.

For (n = 46/80) positive 5-ALA fluorescence, the distribution according to the histological subtype was as follows: adenocarcinoma accounted for 32 (70%) cases, small cell carcinoma for 4 (9%) cases, clear cell carcinoma for 2 (4%) cases, squamous cell carcinoma for 4 (9%) cases, malignant melanoma for 1 (2%) case, large cell carcinoma for 1 (2%) case, and poorly differentiated carcinoma for 2 (4%) cases. In total, 68% (n = 32/47) of adenocarcinomas showed either strong or weak fluorescence intraoperatively. Half of the metastases of small cell carcinoma, 50% (n = 4/8), and clear cell carcinoma, 50% (n = 4/8), tended to fluoresce intraoperatively. More than half of the metastases of squamous cell carcinoma did not show fluorescence. Here, the fluorescence rate was 40% (n = 4/10). Only one quarter of metastases from a malignant melanoma showed fluorescence (n = 1/4). In one metastasis of a large cell carcinoma, 5-ALA fluorescence was observed. The correlation between the 5-ALA fluorescence and the histological subtype is illustrated in Figure 3. Following the statistical analysis, no significant correlation was found between fluorescence behavior and the histological subtype (*p* = 0.518; Chi-square test, SPSS).

### 3.5. Correlation between 5-ALA Fluorescence and Extent of Surgical Resection

**Figure 4:**
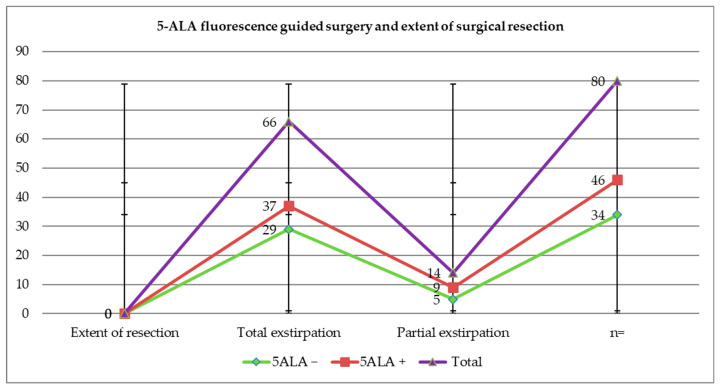
Distribution between 5-ALA fluorescence and extent of surgical resection.

**Figure 5:**
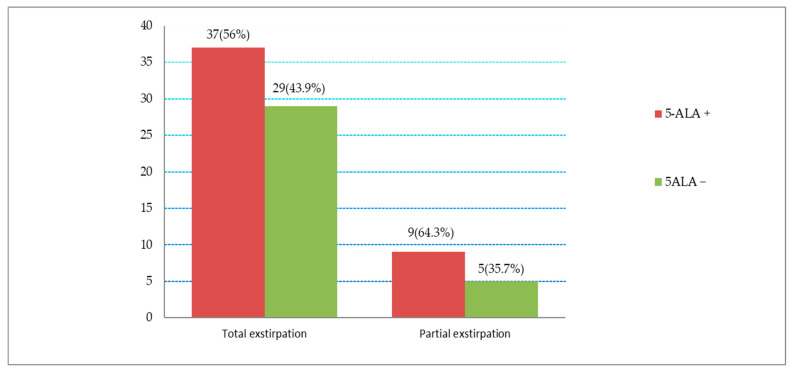
Comparison between total and partial resection in patients with positive and absent 5-ALA fluorescence. Here, it is shown that total extirpation of the metastasis improved by 12.1% in 5-ALA-guided surgery.

Based on radiological criteria, in 17.5% of cases (n = 14/80), a residual tumor was detected (metastasis in eloquent areas or a premature termination of the operation due to electrophysiological changes). However, a complete resection of the brain metastasis was achieved in 82.5% (n = 66/80). In 56% of these (n = 37/66), total resection of the brain metastasis was achieved with the presence of intraoperative 5-ALA fluorescence. However, in (43.9%) (n = 29/66), total extirpation was achieved despite the absence of 5-ALA fluorescence. Information on the distribution between 5-ALA fluorescence and the extent of surgical resection is shown in Figure 4 and Figure 5. Here, the percentage of tumors and the gross total resection rate (GTR) in each category, fluorescent and non-fluorescent, was presented. The t-test analysis resulted in a *p*-value of 0.602, which was not significant. In this study, although the presence or absence of 5-ALA fluorescence did not demonstrate a significant influence on the extent of surgical resection, there was a trend towards improvement in the resectability rate at 12.1% with 5-ALA-guided surgery.

### 3.6. Progression-Free Survival (PFS) and Survival Rate

**Figure 6:**
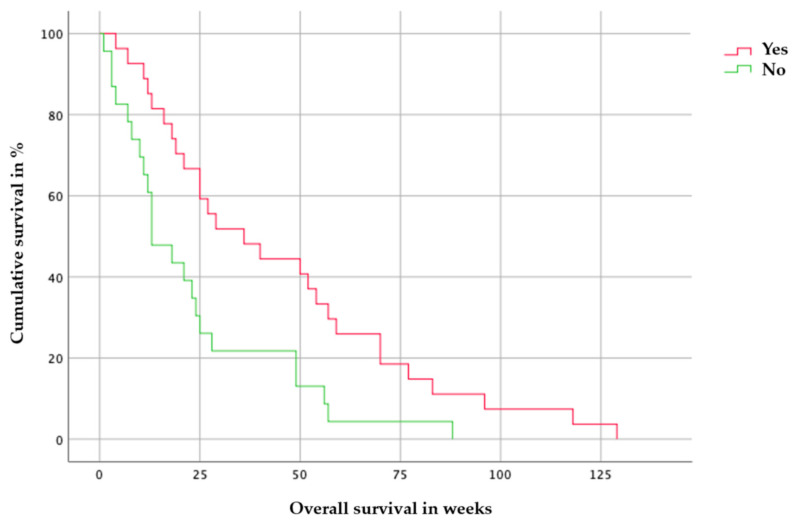
Kaplan–Meier, log-rank test, the figure shows a significant difference in overall survival between 5-ALA-positive (red line) and 5-ALA-negative (green line) metastases. (*p* = 0.009, 95% CI, log-rank test).

Of the 73 patients who underwent 5-ALA-assisted metastases resection, 9 patients (n = 9/73) (12.3%) died in the first eight weeks postoperatively either because of early postoperative complications, postoperative radiotherapy, or because of previous internal diseases. The median survival time for patients with fluorescent metastases was 58 weeks, compared to 20 weeks for patients with non-fluorescent metastases. Fluorescence-positive and -negative metastases showed significantly different overall survival (*p* = 0.009, 95% CI; log-rank test), Figure 6.

**Figure 7:**
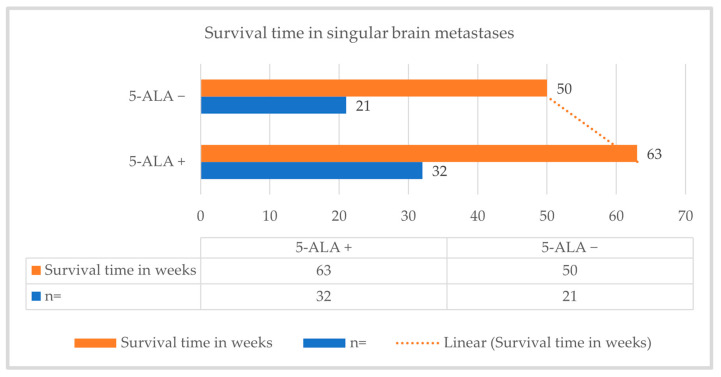
The median survival time of fluorescence-guided and conventionally performed resection of a solitary/singular brain metastasis.

Among the 53 patients who had solitary or singular brain metastases, the median survival time was 63 weeks in those with 5-ALA fluorescence-guided resections and 50 weeks in those with conventional surgical resection (due to absence of fluorescence), which results in a 13-week improvement in median survival in the 5-ALA fluorescence-guided resection of solitary/singular metastases. A correlation was observed between conventional and 5-ALA fluorescence-guided resection of solitary/singular metastases and median survival time (*p* = 0.001), Figure 7.

## 4. Discussion

### 4.1. A Summary of the Results

In the present work, a positive fluorescence rate of 57.5% of all brain metastases was found. Subgroup analysis showed that brain metastases originating from a breast carcinoma had the highest fluorescence rate at 73.3%. In the histological differentiation, adenocarcinomas, regardless of their origin, showed the highest fluorescence at 68.1%. No correlation between fluorescence behavior and localization, primary tumor, or histological subtype could be found. Prior administration of 5-ALA tended to improve the resectability rate by 12.1%. Fluorescence-positive and -negative metastases showed significantly different overall survival. Patients with positive fluorescence of singular metastases showed a significantly prolonged survival time.

### 4.2. Use of 5-Aminolevulinic Acid in Neurosurgery

5-ALA fluorescence-assisted tumor resection continues to gain importance in neurosurgery. While the methodology initially only played a role in glioma surgery of higher-grade tumors, the use of 5-ALA is currently also being investigated for benign tumors such as meningiomas, but also for malignant, metastatic tumors. In the earlier studies, Stummer et al. found in 1998 that after exogenous administration of 5-ALA and due to the defective blood–brain barrier, fluorescent PpIX accumulates in human malignant gliomas and can be visualized intraoperatively in an ultra-violet ambient light of wavelength 375–440 nm [17,18]. Here, a significant advantage of the fluorescence-assisted surgical procedure was observed without the occurrence of noticeable 5-ALA side effects or complications, which led to approval for the use of 5-ALA as a method in the therapy of malignant gliomas [17,27,28]. This makes 5-ALA an established adjunct alongside other auxiliary methods for optimizing tumor resection, such as intraoperative MRI, neuronavigation, or ultrasound. However, these methods should not replace the anatomical knowledge and experience of the surgeon. From previous studies on the use of 5-ALA in metastatic surgery and in the detection of 5-ALA-induced fluorescence of neoplasms of various malignant tumors outside the central nervous system, such as skin, bladder and colorectal cancer, which may be causative for brain metastasis, the resection of a brain metastasis under 5-ALA fluorescence technique can be understood [29,30].

The present retrospective study concluded that 57.5% (n = 46/80) of brain metastases showed 5-ALA-induced fluorescence and 42.5% (n = 34/80) of brain metastases did not fluoresce. The results show similar trends to previous studies: a 2014 study by Marbacher et al. documented positive intraoperative 5-ALA fluorescence in 52% (n = 34/65) of patients with brain metastases [23]. Two studies, albeit with a smaller patient population, even showed a higher 5-ALA fluorescence rate. In 2007, Utsuki et al. found in a series of 11 patients with brain metastases that 82% of cases (n = 9/11) showed 5-ALA fluorescence [25]. Kamp et al. reported positive 5-ALA fluorescence in 62% of metastases in a 2012 study [24]. In various studies, more than half of the metastases showed a positive 5-ALA fluorescence, which appears as either strong or weak fluorescence. The biochemical cause for the different fluorescence behavior in the histologically different brain metastases is not known.

### 4.3. Correlation between 5-ALA Fluorescence and Primary Tumor Entity

Brain metastases from breast carcinoma in this study showed the highest fluorescence ratio, which is in good agreement with the results of Marbacher et al. from 2014, who showed a 5-ALA fluorescence ratio in brain metastases from breast carcinomas of (71%, n = 5/7) [23]. Frei et al. reported that in a case series of 11 patients with breast cancer, all tissue samples from the primary tumor site were fluorescence positive. Here, however, 5-ALA was administered at a dose of 30 mg/kg bw, 3 h preoperatively [31]. The difference between the biological behavior of the primary tumor and the metastatic tissue as well as the 5-ALA dosage could play an important factor in explaining the fluorescence positivity in brain metastases.

While in the present work the fluorescence rate of brain metastases from tumors of the gastrointestinal tract was lower than that of mammary tumors at 66.6% (n = 6/9), Marbacher et al. reported a 5-ALA fluorescence rate of 86% (n = 6/7) in metastases from gastrointestinal adenocarcinomas. However, the statistically limited significance must be taken into account with the overall small patient collective. For the fluorescence rate for brain metastases from lung carcinomas of 56.5% found in this study, inconsistent data were found in the literature. Furthermore, 5-ALA fluorescence was detected in 25% (n = 1/4) of brain metastases from malignant melanoma in our study. However, in a study by Kamp et al. in 2012, brain metastases from malignant melanoma fluoresced in 75% of cases [24], again showing a large difference with a small number of cases.

### 4.4. Correlation between 5-ALA Fluorescence and Histological Subtype

In the histological differentiation, metastases of adenocarcinomas showed fluorescence in 69.5% of the cases, regardless of their origin. The work of Kamp et al. shows a comparable result of 70%, whereas Marbacher et al. found a somewhat lower fluorescence rate of 48%. There were no comparable data for small and clear cell carcinoma metastases, which were positive in 50% of the cases in our work. This leads to the conclusion that adenocarcinomas showed the highest fluorescence rate, followed by small cell and clear cell carcinomas, but no significant correlation between 5-ALA fluorescence and histological subtype could be found. In summary, the studies performed to date, in agreement with our own data, suggest that neither the primary origin of brain metastases nor the histological subtype correlate significantly with intraoperative 5-ALA fluorescence [32].

In astrocytic glioma surgery, visible 5-ALA-induced fluorescence correlated with high MET-PET uptake, along with a high Ki-67 index [33]. As with glioma, the proliferation marker Ki67, which is used as a prognostic marker in various tumors, may also play a role in both 5-ALA fluorescence and as a prognostic marker in histologically different brain metastases, which have already shown a correlation between 5-ALA fluorescence rate and prolonged survival in our study. However, the biochemical, molecular biological, and cytogenetic differences between brain metastases of different subtypes and gliomas may be causative for the different fluorescence behavior. Certainly, larger patient numbers, and probably in the future, an expansion of data collection or specific gene mutations, mutations of the EGFR gene, detection of the EML4-ALK translocation, and further molecular genetic expression profiles, should be provided. For example, adenocarcinomas of the lung can be typed and classified more precisely not only histopathologically, but also with the help of molecular analyses, which is important for prognosis and therapy response [34]. Different studies have shown a significantly higher fluorescence rate in adenocarcinomas than in other subtypes. Therefore, more targeted studies should be conducted on the correlation between 5-ALA fluorescence and adenocarcinoma with different histomorphological and genetic subtypes.

### 4.5. Influence on Resectability

The extent of surgical resection in this work was predominantly assessed by early postoperative MRI of the skull. Complete resection was observed in 82.5% of cases. In other studies, a complete resection of the metastasis could be observed in 56% of the cases after an MRI examination within the first three days. However, here the extent of the resection could not be precisely determined in 26.1% of the cases [32]. No significant correlation between fluorescence and resection extent could be noticed in our study as well as in other studies [32].

Although the positivity or negativity of 5-ALA fluorescence did not show a significant influence on the degree of surgical resection in this study or in other studies [32], 5-ALA fluorescence-guided surgery in glioblastoma surgery was able to achieve a more radical tumor resection compared to conventional techniques, resulting in increased progression-free survival [17,35]. Here, however, the different fluorescence properties of glioblastomas and metastases, which is based on different biological behavior, and the surgically different characteristics of metastases, which are not intra-axial or infiltrative, should be taken into consideration. 5-ALA fluorescence-guided surgery can still be useful for metastatic surgery. Yoo et al. reported in 2009 that better local tumor control could be achieved by extending the resection of intracerebral metastases, especially in non-eloquent areas, to a depth of about 5 mm [36]. However, sufficient identification of peritumoral infiltrating cells is not always possible even with 5-ALA. There may be non-specific leakage of PpIX and fluorescence in the peritumoral oedematous brain tissue, which may be due to the infiltrating pincer-like growth pattern of a brain metastasis into the adjacent brain tissue [37] on the one hand, and to destruction of the blood–brain barrier in large brain metastases on the other.

### 4.6. Influence on Median Survival Time

Fluorescence-positive and -negative metastases showed significantly different overall survival in this study (*p* = 0.009). In contrast, the work of Kamp et al. had shown that 5-ALA fluorescence behavior had no significant effect on overall survival (*p* = 0.852) [32]. However, in a 2019 paper with a larger patient population, Kamp et al. demonstrated significantly different overall survival between positive and -negative 5-ALA fluorescence in metastatic disease [38]. One possible explanation for a correlation between 5-ALA fluorescence-guided metastasectomy and the prolonged median survival time in our study could be that 5-ALA fluorescence-guided surgery, albeit not statistically significant, tended to achieve more total extirpation of the metastasis than conventional surgery (Figure 5, 12.1% improvement in total extirpation of the metastasis in 5-ALA-guided surgery). Moreover, it may well be that the fluorescent metastases have other histogenetic properties that play a role in 5-ALA uptake and improve prognosis overall. Here, further detailed histogenetic and molecular investigation into their influence on 5-ALA uptake in tumor cells may be advisable in the future. In a study from 2019, 5-ALA fluorescence behavior could be considered as a prognostic marker and was found to correlate significantly with local progression-free and overall survival time [38].

Furthermore, the good prognosis and the prolonged median survival time do not only depend on the completeness of the metastasis resection. The effectiveness of local therapy of the brain metastasis is not the only decisive factor for the median survival time; Soffietti et al. described in a 2006 study that the majority to even more than half of the patients did not die from the brain metastases themselves, but from the consequences of systemic tumor progression [39], which may be the explanation for the prolonged median survival time with fluorescent metastases, despite the lack of statistically significant correlation between 5-ALA fluorescence and the extent of resection of the metastasis. Here, it seems that the fluorescence properties of the metastases not only tend to have an influence on the extent of resection, but that the fluorescence itself has the function of a histological prognostic marker of the metastasis subtype. The value of 5-ALA fluorescence should be investigated in patients with the same primarius and histological subtype and possibly similar stage of systemic cancer.

In brain metastases, it is still much more difficult to understand why some metastases fluoresce and others do not, as we have to take into account different factors that may influence fluorescence. One difficulty for intraoperative fluorescence diagnosis, however, is that the subjective assessment is highly dependent on the surgeons. Here, spectroscopic measurements of the PpIX concentration in tumor tissue could be helpful to digitally measure the strength of fluorescence. This was investigated by Schwake et al. who, in a paper from 2019, showed in eleven children with different types of brain tumors that in all tumors with visible fluorescence, the PpIX concentration exceeded 4 µg/mL. In contrast, PpIX concentrations without visible fluorescence remained below 0.20 μg/mL [40]. This shows that the fluorescence intensity is dependent on the PpIX concentration, which in turn may be related to the density of the tumor cells. This density can also be helpful to better detect the invasiveness of the tumor cells in the surrounding tissue.

The spectroscopic analysis of fluorescence in glioblastomas performed so far may also be helpful in brain metastases, on the one hand to better determine the fluorescence intensity and on the other hand to spectroscopically distinguish 5-ALA fluorescence in the tumor tissue from 5-ALA leakage in the peritumoral tissue. In addition to intraoperative imaging to optimize tumor resection, 5-ALA as a natural biochemical precursor of hemoglobin, which causes the synthesis and accumulation of highly fluorescent PpIX in malignant glioma tissues, is a good aid for complete resection of a tumor [37], but its value in metastatic surgery cannot yet be conclusively assessed.

### 4.7. Biochemical Laboratory Parameters

Significant but not clinically relevant leukocytosis, thrombocytopenia and anemia were found in the study in accordance with the Gliolan technical information [41]. The extent to which the anemia was 5-ALA-induced, bleeding-associated or dilated cannot be correlated, nor can the leukocyte increase, which could also be due to dexamethasone. The postoperative increase in the hepatic parameter GPT was also not significantly changed, as also described in the literature [32], but there was a significant, but not clinically relevant, increase in g-GT [42]. The postoperative creatinine values as renal parameters were significantly lower, which could possibly be due to the increased perioperative fluid measurements; there was no evidence of renal dysfunction. Overall, no permanent side effects were observed with 5-ALA during the inpatient stay. Analogous to the use of 5-ALA in glioma surgery, there were no indications of 5-ALA-associated toxicity. Thus, these data do not speak against the use of 5-ALA in neurosurgery.

Regarding the dosage, the different fluorescence behavior of glioblastomas and metastases could be caused by a different biological behavior, so that the higher dosage of 30 mg/kg bw, as applied in the study by Frei et al., could lead to an improved fluorescence of metastases. In the future, the dosage and the time of preoperative administration of 5-ALA could be investigated in a more differentiated manner, because different dosages and/or periods of administration would be conceivable with different proliferation rates of the metastatic cells.

### 4.8. Critical Discussion of the Limitations of This Study, Which Was Designed as a Retrospective Study

- One difficulty in the fluorescence diagnosis (absent, weak, or strong) was that its evaluation was very subjective depending on the surgeons. Here, to avoid subjectivity, spectroscopic measurement of PpIX concentration could be useful in the future, which has already been investigated by Kaneko et al. in a 2019 paper. Here, using a hyperspectral camera, they were able to demonstrate that both fluorescence intensity and PpIX concentration were higher centrally than peripherally in high-grade gliomas. They also observed, interestingly, that the marginal weak fluorescence peaked later than the central strong fluorescence (8–9 vs. 7–8 h) [43].
- To observe the long-term laboratory chemical change after 5-ALA administration, laboratory controls over time were lacking. The dosage of 5-ALA used in this work referred to the previously recommended 5-ALA dosage in glioblastoma surgery. Due to different tumor cell entities of the metastases, higher 5-ALA doses could be targeted.
- In this retrospective work, the interoperative adjuvant methods (such as neuromonitoring, neuronavigation, or ultrasound) were not regularly used, which could influence the intraoperative assessment of complete resection of the metastasis.
- The adenocarcinomas could be typed and classified more precisely after molecular analyses; here, more targeted investigations should be carried out for a possible correlation between the 5-ALA fluorescence and the adenocarcinoma with different histomorphological and genetic subtypes. In order to improve the study quality, based on the present retrospective design, the study protocols should be expanded accordingly in a future prospective study and the data collections should be performed in a larger patient population, taking into account the histomorphological and genetic subtypes of brain metastases.

## 5. Conclusions

In this study, a fluoresce rate of brain metastases at 57.5% was found. Subgroup analysis showed that brain metastases originating from a breast carcinoma had the highest fluorescence rate at 73.3%. In the histological differentiation, adenocarcinomas, regardless of their origin, showed highest fluorescence at 68.1%. No correlation between fluorescence behavior and localization, primary tumor, or histological subtype could be found. Prior administration of 5-ALA tended to improve the resectability rate by 12.1%. Fluorescence-positive and -negative metastases showed significantly different overall survival. Patients with positive fluorescence of singular metastases showed a significantly prolonged survival time. Therefore, administration of 5-ALA as a surgical adjuvant may be beneficial in resecting brain metastases and may potentially optimize the extent of resection in modern surgery for brain metastases.

## Figures and Tables

**Table 1:** Graded Prognostic Assessment (GPA) score.

Parameter	Score = 0	Score = 0.5	Score = 1
age (yrs)	>60	50–59	<50
KPS score	<70	70–80	90–100
number of CNS metastases	>3	2–3	1
extracranial metastases	yes		no

Prognosis classes of (GPA): The median survival times were 2.6 months for GPA 0–1, 3.8 months for GPA 1.5–2.5, 6.9 months for GPA 3, and 11.0 months for GPA 3.5–4.

**Table 2:** Possible reasons for tumor-selective PpIX accumulation.

Parameter	Source of Selectivity	Effect
1	Blood brain barrier leakage in glioma	Normal brain fairly protected from 5-ALA
2	Increased activity of GABA, pepT1, pepT2 transporters	Uptake of 5-ALA in glioma cells increased
3	Increased activity of ALA-D	More of PpIX-precursor PBG synthesized
4	Increased activity of PBG-D	More of PpIX-precursor HMB synthesized
5	Reduced ferrochelatase activity	Accumulation of PpIX as one of the substrates of this step
6	Reduced availability of Fe^2+^	Accumulation of PpIX as the other substrate of this step
7	ABCB6-transporter	Transport of CPgen III into mitochondria
8	ABCG2-transporter	Transport of PpIX from mitochondria into the cytosol, but also loss of PpIX through the plasma membrane

ALA-D: ALA-dehydrogenase; PBG: Porphobilinogen; PBG-D: Porphobilinogen-deaminase; HMB: hydroxymethylbilane; CPgen III: coproporphyrinogen III, modified form.

**Table 3:** Disease- and patient-related data. From the primary tumor onwards, both the numbers and percentages were calculated on the basis of cases (not patients).

Parameter	n (%)
number of patients	73
number of cases	80
age:	
Mean	63 years
age range	33–83 years
gender:	
Female	35 (47.9%)
Male	38 (52.1%)
primary tumor:	
bronchial carcinoma	46 (57.5%)
breast carcinoma	15 (18.8%)
gastrointestinal carcinoma	9 (11.3%)
renal cell carcinoma	5 (6.3%)
malignant melanoma	4 (5.0%)
gynecological carcinoma	1 (1.3%)
Histology:	
Adenocarcinoma	47 (58.7%)
squamous cell carcinoma	10 (12.5%)
small cell carcinoma	8 (10.0%)
malignant melanoma	4 (5.0%)
clear cell carcinoma	4 (5.0%)
giant cell carcinoma	2 (2.5%)
poorly differentiated brain metastases from carcinoma	5 (6.3%)
localization of the metastasis:	
Supratentorial	60 (75.0%)
Infratentorial	17 (21.2%)
supra/infratentorial	3 (3.8%)
eloquent area	39 (49.0%)
non-eloquent area	41 (51.0%)
number of metastases:	
singular	58 (72.6%)
multiple	22 (27.4%)
5-ALA fluorescence:	
positive	46 (57.5%)
negative	34 (42.5%)
degree of surgical resection:	
total	66 (82.5%)
partial	14 (17.5%)
incidence of complete resection of the metastasis:	
total extirpation	66 (82.5%)
5-ALA positive	37 (56.1%)
5-ALA negative	29 (43.9%)
eloquent area	31 (46.9%)
non-eloquent area	35 (53.1%)

## Data Availability

Data are contained within the article.
